# Empathy and stress related neural responses in maternal decision making

**DOI:** 10.3389/fnins.2014.00152

**Published:** 2014-06-12

**Authors:** S. Shaun Ho, Sara Konrath, Stephanie Brown, James E. Swain

**Affiliations:** ^1^Department of Psychiatry, University of MichiganAnn Arbor, MI, USA; ^2^Research Center for Group Dynamics, Institute for Social Research, University of MichiganAnn Arbor, MI, USA; ^3^Department of Psychiatry, University of Rochester Medical CenterRochester, NY, USA; ^4^Department of Psychiatry and Behavioral Science, Stony Brook UniversityNew York, NY, USA

**Keywords:** empathy, cortisol, amygdala, hypothalamus, functional MRI, mothers, decision making, social neuroscience

## Abstract

Mothers need to make caregiving decisions to meet the needs of children, which may or may not result in positive child feedback. Variations in caregivers' emotional reactivity to unpleasant child-feedback may be partially explained by their dispositional empathy levels. Furthermore, empathic response to the child's unpleasant feedback likely helps mothers to regulate their own stress. We investigated the relationship between maternal dispositional empathy, stress reactivity, and neural correlates of child feedback to caregiving decisions. In Part 1 of the study, 33 female participants were recruited to undergo a lab-based mild stressor, the Social Evaluation Test (SET), and then in Part 2 of the study, a subset of the participants, 14 mothers, performed a Parenting Decision Making Task (PDMT) in an fMRI setting. Four dimensions of dispositional empathy based on the Interpersonal Reactivity Index were measured in all participants—Personal Distress, Empathic Concern, Perspective Taking, and Fantasy. Overall, we found that the Personal Distress and Perspective Taking were associated with greater and lesser cortisol reactivity, respectively. The four types of empathy were distinctly associated with the negative (vs. positive) child feedback activation in the brain. Personal Distress was associated with amygdala and hypothalamus activation, Empathic Concern with the left ventral striatum, ventrolateral prefrontal cortex (VLPFC), and supplemental motor area (SMA) activation, and Fantasy with the septal area, right SMA and VLPFC activation. Interestingly, hypothalamus-septal coupling during the negative feedback condition was associated with less PDMT-related cortisol reactivity. The roles of distinct forms of dispositional empathy in neural and stress responses are discussed.

## Introduction

Parents make numerous daily choices regarding how to best care for their children. Parents must make quick decisions and learn from their child's feedback to guide their next course of action. Unfortunately, children may not always provide predictable, desirable feedback to guide parental responses. To make matters worse, responses that were effective at one time may not be effective at another time, leaving children upset or in need. Such unpredictable negative feedback may augment frustration in both parents and children and undermine a healthy parent-child relationship in the long run, thus highlighting the need for high parental sensitivity and attunement (Feldman et al., 2004; Swain et al., 2014).

Sensitive parents must be able to empathically tolerate the stress of negative feedback, and it is likely that this negative feedback from children does not impact all parents equally. Indeed, parental sensitivity to children's needs is related to parents' own developmental history, resources, and their notions and dispositions related to child rearing (Cox and Harter, 2003; Shin et al., 2006; Leerkes, 2010). Moreover, research finds that people differ in their dispositional empathy in response to other people's distressing experiences. Indeed, empathy is one of the most important dispositions in interpersonal relationships and social wellbeing (Davis, 1996). Given this, it is not surprising that empathy is critical to sensitive parenting (Feshbach, 1990; Davidov and Grusec, 2006; Landry et al., 2006; Psychogiou et al., 2008).

### Dispositional empathy and stress reactivity

Empathy is a disposition that is relatively stable across the lifespan (Konrath, under review). As construed in Davis' Interpersonal Reactivity Index (IRI) (Davis, 1980, 1983), empathy can be parsed into four dimensions. Perspective-Taking (PT) assesses the tendency to spontaneously adopt the psychological point of view of others. Empathic Concern (EC) assesses feelings of compassion and concern for unfortunate others. Fantasy (FS) assesses respondents' tendencies to transport themselves imaginatively into the feelings and actions of fictional characters. Personal Distress (PD) assesses “self-oriented” feelings of personal anxiety and unease in response to others' tense experiences (Davis, 1980). Empathic concern and personal distress are both affective but they can impact people's social behaviors differently. For example, empathic concern usually promotes prosocial behaviors but personal distress often hinders prosocial behaviors, potentially due to self-oriented anxiety elicited by others' suffering (Eisenberg, 2000).

Physiologically, these distinct emotional components of empathy may alter stress responses in opposite directions. There is indeed some evidence that while empathic concern may reduce cortisol reactivity to stressful situations, personal distress may elevate such responses. Consistent with the Caregiving Model of Stress Regulation (Swain et al., 2011, 2012, 2013; Brown et al., 2012; Konrath and Brown, 2013), one experiment demonstrated that participants who gave social support to a stressed partner experienced declines in cortisol levels during the experiment (Smith et al., 2009). Although giving support is not identical to empathic concern, the pattern of findings supports a notion that focusing on another's needs may help an individual attenuate stress responses. A similar study examined the cortisol responses of participants who completed the standard Trier Social Stress Task (job interview speech) compared to those who also gave a job interview speech, but were asked to focus on how they could help others with the job (Abelson et al., 2014). Participants in the compassion condition showed attenuated cortisol responses during this stressful task. Conversely, dispositional low empathy (i.e., narcissism) has been linked to significantly elevated cortisol levels overall (Reinhard et al., 2012) and in response to stressors (Edelstein et al., 2010), especially among males.

Despite this work, no research that we are aware of directly examines how the brain may mediate different cortisol responses in the context of empathy. In the current study, we examined how the four distinct empathy constructs play a role in cortisol-related stress responses in two different potentially stressful social contexts, being evaluated by others (Part I) and failing to meet a child's needs (Part II).

### Dispositional empathy in the brain

In Part II of the current study, we also examine whether exposure to distressed children differentially activates brain areas as a function of the four dimensions of dispositional empathy. Key neural regions of interest were retrieved from three recent meta-analyses on neural activations associated with empathy (Seitz et al., 2006; Fan et al., 2011; Lamm et al., 2011), which include the ventromedial prefrontal cortex (VMPFC), ventral anterior cingulate cortex (VACC), dorsol ACC (DACC), anterior middle cingulate cortex (AMCC), supplemental motor area (SMA), ventrolateral prefrontal cortex (VLPFC), superior temporal gyrus, anterior insula, parietal lobes, and precuneus. In addition, the septal area, which is involved in maternal caregiving-related defense (D'Anna and Gammie, 2009) and stress regulation (Singewald et al., 2011), has been found to be associated with empathy across social contexts (Morelli et al., 2014). While these regions of interest are commonly activated in empathy-inducing tasks (e.g., observing cues or pictures of suffering from self's or other's perspective), the distinct roles of the four dimensions of dispositional empathy in these neural responses have not been examined in simulated interpersonal interactions (e.g., between mother-child).

### Stress reactivity in the brain

In Part II, we used a maternal decision task to evaluate the influence of positive or negative feedback from a child on neural responses related to stress (e.g., activation of the amygdala, hypothalamus), the stress hormone cortisol, and the four types of dispositional empathy. Cortisol reactivity is the sequela of the limbic-hypothalamus-pituitary-adrenal axis (LHPA-axis) response (Feldman et al., 1995; Wilkinson and Goodyer, 2011), and the amygdala is the primary limbic structure in the LHPA-axis that has been shown in animal models to initiate parenting neural circuitry, triggering the motivation for parenting by activating sub-nuclei in the hypothalamus (Feldman et al., 1995; Dayas et al., 1999). The recent social neuroscience literature has suggested that the amygdala plays a key role in maternal sensitivity in humans and these functional brain activities may be linked to stress-modulating hormones (Atzil et al., 2011).

## Materials and methods

### Procedure

Participants completed the Interpersonal Reactivity Index (IRI) before the brain scan. They then underwent a 6 min Social Evaluation Test (SET; Wager et al., 2009) after they were randomly assigned to either a social interaction condition or a control condition (data to be reported elsewhere), with salivary cortisol measured pre-SET (15 min before) and post-SET (15 min after). On a different day,on average 7 days later, 14 participants (all mothers) returned to undergo a Parental Decision Making Task (PDMT). Salivary cortisol samples were collected pre-PDMT (15 min before) and post-PDMT (about 15 min after). All procedures were approved by University of Michigan's Institutional Review Board.

### Participants

Participants were 33 mentally and physically healthy women (16 mothers and 17 non-mothers, mean age = 29.06, *SD* = 6.77). They all completed the SET (Part 1) and only 14 of those mothers completed the PDMT in Part 2, with a mean age = 32.86, *SD* = 6.54, 1–5 children (mean number = 1.93, *SD* = 1.07; mean children's age = 3.90, *SD* = 3.27).

### Measures

The Interpersonal Reactivity Index (IRI; Davis, 1980) consists of 28 items and measures four dimensions of empathy: Perspective Taking (PT, e.g., “I try to look at everybody's side of a disagreement before I make a decision”), Fantasy (FS, e.g., “I really get involved with the feelings of the characters in a novel”), Empathic Concern (EC, e.g., “ I often have tender, concerned feelings for people less fortunate than me”), and Personal Distress (PD, e.g., “I sometimes feel helpless when I am in the middle of a very emotional situation”). Each dimension is composed of 7 items (1 = does not describe me well; 5 = describes me very well).

### Salivary cortisol

To collect salivary cortisol, participants were asked to provide passive drool samples during two data collection times: pre- and post-task. Salivary cortisol levels were determined by chemiluminescent enzyme immunoassay (IMMULITE) according to the manufacturer's directions (Siemens Healthcare Diagnostics Inc., Tarrytown, NY).

### Social evaluation test

We used similar SET procedures as described in Wager et al. (2009). There were three phases that were each 2 min long: Baseline, Speech Preparation, and Relaxation. After the 2 min resting period (baseline), participants were given 2 min to prepare a 7-min speech on “Why I am a good friend” (Speech Preparation), which they were told might be recorded and evaluated for its quality and organization by experts. However, after the preparation period, all participants were told that the speech was no longer needed and that they could relax for the 2 min (Relaxation).

### Parenting decision making task

**Figure 1:**
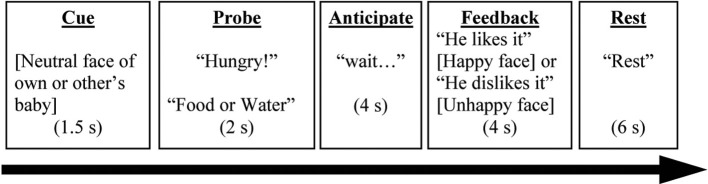
**A complete trial in the Parenting Decision Making Task is depicted here**. The events labels (underscored) were not presented on the screen during the task. Words in brackets referred to a picture of a child. Words in quotation were the texts presented during the task.

The PDMT was designed to probe brain circuits underlying goal-directed parenting behaviors in the context of parent-child interactions. The stimuli consisted of pictures of four different children of the same sex, including one who was the participant's own child and three who were unknown to participants, acquired from a commercial source. Each of these four children was presented with three pictures, one each of neutral, happy, and unhappy expression, in the task. Thus, stimuli consisted of 12 pictures total (3 pictures × 4 children). Before the brain scan, participants were shown pictures of the three unknown children to reduce novelty effects. During the task, participants were instructed to attend to the child's need. In each trial, one of the children's neutral expression pictures was first presented on the screen for 1.5 s (Cue). Following this, a probe “Hungry” or “Thirsty” along with choices “Food” or “Water” were presented for up to 2 s (Probe). Participants were instructed to press a button corresponding to “Food” or “Water” to match the probe correspondingly. As soon as the button-pressing response was made, an anticipation period with “waiting for his/her reaction… ” was shown on the screen for 4 s (Anticipation). Each trial concluded with a 4-s feedback phase showing positive (i.e., child's happy face) or negative (i.e., child's unhappy face) feedback, along with the written outcome “He/she is happy (or unhappy)!” respectively (Feedback). The inter-trial interval (Rest) was 6 s. For the neuroimaging results, we focused on the feedback phase differentiating Positive Feedback (happy face) and Negative Feedback (unhappy face) in the current study. See Figure 1.

Unbeknownst to the participants, the valence of a child's feedback was randomly selected based on a pre-determined probability. For the participant's own child, the probability was 50% for positive and negative feedback. For the three unknown children, the probabilities of the positive feedback were 75% (for an “easy” child), 50% (for an “ambiguous” child), and 25% (for a “difficult” child). In the current study, we only examined the neural responses to positive and negative feedback across all four children in fMRI analyses, because we aimed to identify the effects of dispositional empathy that could be generalized across different children.

To dissociate the brain-imaging signals related to different components of the trials (Ollinger et al., 2001), a number of partial trials, e.g., (Cue), (Cue + Probe), or (Cue + Probe + Anticipation) were randomly interwoven with the complete trials throughout the task (12 trials per type of partial trials). These were in addition to the complete trials (12 trials per child type; 48 trials total). The tasks were divided into three runs of 6.5 min each.

### Behavioral data analysis related to pdmt

The accuracy and reaction time (RT) of the binary choice response (“food” or “water”) when prompted with a probe (“Hungry” or “Thirsty”) were analyzed using repeated measures analyses, using the child types as a within-subject independent variable. Age and the four empathy subscales were entered as between-subjects covariates.

### fMRI data acquisition and preprocessing

Scanning took place in a 3.0 Tesla Philips magnetic resonance imaging scanner with a standard 8-channel SENSE head coil. Functional data was acquired (300 T2^*^-weighted EPI volumes, *TR* = 2000 ms, *TE* = 30 ms, flip angle = 90, field of view = 220 mm, matrix size 64 × 64, 42 axial slices, voxels = 3.44 × 3.44 × 2.80 mm). A high-resolution anatomical T1-weighted image with a three dimensional gradient recalled echo was also acquired with *TR* = 9.8 ms, *TE* = 459 ms, *FA* = 8°, FOV = 256 mm, 180 slices with 288 × 288 matrix per slice, 1 mm slice. Five images at the beginning of each fMRI run were discarded to account for magnetic equilibrium. Functional imaging data were preprocessed and analyzed using SPM8 (Statistical Parametric Mapping 8; Wellcome Trust Center for Neuroimaging, University College, London, UK; http://www.fil.ion.ucl.ac.uk/spm). Slice timing correction was performed using a middle slice as a reference (slice 21). After slice time correction, images within each run were realigned to the first image of the first run to correct for movement. Realigned functional images and structural image were spatially normalized using DARTEL method in SPM8. The normalized functional images were re-sliced to 2 × 2 × 2 mm voxels. Images were then spatially smoothed using a Gaussian filter with a full-width half-maximum value of 8 mm.

### fMRI data analysis

At the individual subject level, response amplitudes were estimated for each condition using the general linear model. A high pass filter of 0.0078 Hz (1/128 s) was used. Seventeen distinct events in the task were modeled, except resting period, including Cues × 4 (one per child type), Probe, Anticipation × 4 (one per child type), Positive Feedback (one per child type) and Negative Feedback (one per child type). For individual subjects, we contrasted images of the blood oxygen level-dependent (BOLD) signal change associated with Negative vs. Positive Feedback (all four children combined) as the contrast of interest.

To examine the relationship between event-related activity in the hypothalamus and task-related salivary cortisol change, a functional connectivity analysis was performed at the individual subject level as well. Here we focused on the negative feedback across all types of children. In this analysis, the hypothalamus as the seed was defined as a rectangular volume bounded within a range of MNI coordinates of (*x* = −8~8, *y* = −8~0, *z* = −4~−16]. The physiological variable was estimated to be the average of the first eigenvariate of the BOLD time series of all voxels in the hypothalamus seed throughout the task. Then, this physiological variable is parsed into 17 event-specific time-series based on the time window of 17 modeled events, defined by the onset and duration of each type of event convolved with the canonical hemodynamic response function. Then, the whole time series of the hypothalamus seed, the 17 event-related time series of the hypothalamus seed, the 17 events modeled as in a regular event-related design, and 6 motion parameters estimated during the realignment preprocessing were all entered in a general linear model to perform a generalized psychological-physiological interaction analysis (gPPI) (Mclaren et al., 2012).

For the group-level analysis, the Negative vs. Positive Feedback contrast images for individual subjects were entered into random-effects GLM analyses, with age and PD, PT, EC, or FS used as the predictors. A priori regions of interest (ROI) were those that are known to be associated with face-based reward in a social context (Ho et al., 2012), empathy-related neural regions (Seitz et al., 2006; Fan et al., 2011; Lamm et al., 2011), and the stress system, including the hypothalamus, amygdala, ventral striatum, VACC, AMCC, anterior insula, SMA, VLPFC, VMPFC, and precuneus. They were defined by the anatomical masks adapted from WFU pickatlas toolbox (http://fmri.wfubmc.edu/cms/software), wherein statistical maps in these regions were small volume corrected at a thresholded of *p* = 0.05 with family-wise correction.

Note that while the scans took place at different time of the day (*n* = 8 in the morning and *n* = 6 in the afternoon), which may influence the pre-task cortisol baseline, time of day was not associated with cortisol reactivity, dCORT, defined as the difference between post- and pre-PDMT cortisol levels (*p* = 0.456). Still, any potential confounding was addressed by including the time of scan as a covariate in the cortisol analyses described above.

To identify neural correlates of PDMT-related cortisol reactivity, the analyses were conducted in two steps. In the first step, in SPM8, the individual-specific Negative vs. Positive Feedback contrast images (across all child types) were submitted to a regression model that contained the difference between post-task and pre-task salivary cortisol levels (dCORT) as a single regressor. If a cluster was found to be significantly associated with the dCORT in ROIs that survived small volume corrections, the averaged parameter estimates of that cluster were computed for each subject and used in the next step. In the second step, using IBM SPSS 21, the partial correlations between the cluster's parameter estimates and dCORT were computed, controlling for age and time of scan (morning or afternoon). The same two-step approach was utilized for the functional connectivity analysis, using the hypothalamus as the seed to identify clusters within the a priori ROIs that were coupled with the hypothalamus during negative feedback across all child types.

## Results

### Part 1

Pre-SET cortisol levels were at 0.19 mcg/dL (*SE* = 0.019) and post-SET levels were at 0.18 mcg/dL (*SE* = 0.16), controlling for between-subject variables of maternity status (mothers or non-mothers), prior social interaction condition, age, and time of cortisol collection (binary, morning or afternoon). To examine the relationship between the SET-related change in cortisol (dCORT) and the four dimensions of dispositional empathy (i.e., Personal Distress, PD; Empathic Concern, EC; Perspective Taking, PT; and Fantasy, FS), partial correlations among these variables were computed, controlling for age, binary coding for the time of cortisol measurement (morning or afternoon), maternal status (mothers or non-mothers), and the randomly assigned pre-SET condition (social interaction or none). The results are summarized in Table 1.

**Table 1:** **Pairwise partial correlations (Pearson's *r* with *p*-values in parentheses) between cortisol reactivity (post-task minus pre-task CORT, denoted as dCORT) and dispositional empathy, controlling for age, maternal status, time of Social Evaluation Test, and pre-test manipulation (*n* = 33 women)**.

	**dCORT**	***PT***	***EC***	***FS***	***PD***
dCORT	1				
*PT*	−0.40^*^ (0.030)	1			
*EC*	−0.017 (0.93)	0.62^***^ (0.001)	1		
*FS*	0.065 (0.74)	−0.045 (0.82)	0.18 (0.35)	1	
*PD*	0.48^**^ (0.009)	−0.28 (0.14)	−0.041 (0.83)	0.079 (0.68)	1

* *Correlation is significant at the 0.05 level (2-tailed)*.

** *Correlation is significant at the 0.01 level (2-tailed)*.

*** *Correlation is significant at the 0.001 level (2-tailed)*.

Notably, SET-induced cortisol reactivity was positively associated with Personal Distress (PD), while it was inversely associated with Perspective Taking (PT) (see Table 1, Column 1). These results suggest a linkage between dispositional empathy and stress responses. Although these results were specifically found with respect to socially evaluative situations, they may possibly be generalized to other mildly stressful contexts such as the simulated parenting context from Part 2 of the current study.

### Part 2

Only the results from the participants (*n* = 14) who were mothers and underwent the PDMT during the fMRI session are included henceforth. In this smaller subsample, the descriptive statistics of the IRI scores were as follows (mean, with standard deviation in parentheses): *PT* = 3.45 (0.55), *EC* = 3.84 (0.62), *FS* = 2.98 (1.08), and *PD* = 1.73 (0.92). In accordance with the larger samples reported above, controlling for age, PT and EC were still significantly correlated (*r* = 0.63, *p* = 0.02). However, no correlations were found between any other pairs of dispositional empathy subscales, PT-PD (*r* = −0.060, *p* = 0.85), PT-FS (*r* = 0.045, *p* = 0.89), EC-FS (*r* = 0.47, *p* = 0.11), EC-PD (*r* = 0.094, *p* = 0.76), and FS-PD (*r* = 0.28, *p* = 0.35).

Pre-PDMT cortisol levels were at 0.21 mcg/dL (*SE* = 0.031) and post-PDMT levels were at 0.15 mcg/dL (*SE* = 0.011), controlling for age and time of cortisol collection (binary, morning or afternoon). To examine the relationship between the SET-related changes in cortisol (dCORT) and the four dimensions of dispositional empathy, partial correlations among these variables were computed, controlling for age and binary coding for the time of cortisol measurement (morning or afternoon). The results are summarized in Table 2.

**Table 2:** **Pairwise partial correlations (Pearson's *r* with *p*-values in parentheses) between cortisol reactivity (post-task minus pre-task CORT, denoted as dCORT) and dispositional empathy, controlling for age and time of Parental Decision Making Task (*n* = 14)**.

	**dCORT**	***PT***	***EC***	***FS***	***PD***
dCORT	1				
*PT*	0.21 (0.52)	1			
*EC*	0.20 (0.54)	0.57^#^ (0.053)	1		
*FS*	0.29 (0.36)	0.30 (0.35)	0.69^*^ (0.013)	1	
*PD*	−0.067 (0.84)	−0.055 (0.87)	0.11 (0.74)	0.30 (0.35)	1

# *Correlation is significant at the 0.10 level (2-tailed)*.

* *Correlation is significant at the 0.05 level (2-tailed)*.

These results suggested that the functional MRI task did not elicit a significant stress response and that cortisol reactivity was not associated with any of the four dimensions of dispositional empathy. A significant correlation between Empathic Concern and Fantasy, and a marginally significant correlation between Perspective Taking and Empathic Concern were found in this smaller sample. The discrepancy between the SET and PDMT results may be attributed to the differences in the nature of the tasks and the sample size.

### PDMT behavioral results

We next conducted a repeated measurement general linear model examining the accuracy and RT for each child type when the participants chose responses (“food” or “water”) when prompted with a probe (“Hungry” or “Thirsty”) during the Parenting Decision Making Task. Child type was the within subject variable and age and the four dimensions of dispositional empathy were covariates. The descriptive statistics of accuracy and RT for the own child (50% probability of positive feedback): mean accuracy = 0.92, *SE* = 0.021, and mean *RT* = 842.1 ms, *SE* = 78.6; for the ambigious other child (50% probability of positive feedback): mean accuracy = 0.90, *SE* = 0.026, and mean *RT* = 818.4 ms, *SE* = 83.5; for the difficult other child (25% probability of positive feedback): mean accuracy = 0.90, *SE* = 0.026, and mean *RT* = 895.8 ms, *SE* = 56.3; and for the easy other child (75% probability of positive feedback): mean accuracy = 0.90, *SE* = 0.036, and mean *RT* = 854.7 ms, *SE* = 48.7. There were no main effects of child type on this behavioral performance. Neither accuracy nor RT differed as a function of child type [Accuracy: *F*_(3, 24)_ = 0.21, *MS*_error_ = 0.003, *p* = 0.89, *N.S*; *RT*: *F*_(3, 24)_ = 0.23, *MS*_error_ = 12690.4, *p* = 0.872, *N.S*].

For the between-subject factors, age was inversely associated with accuracy [*F*_(1, 8)_ = 22.29, *MS*_error_ = 0.034, *p* = 0.001], but not associated with *RT* [*F*_(1, 8)_ = 0.25, *MS*_error_ = 223590.39, *p* = 0.63, *N.S*]; Fantasy was associated with accuracy [*F*_(1, 8)_ = 6.88, *MS*_error_ = 0.034, *p* = 0.031), but not with RT [*F*_(1, 8)_ = 0.16, *MS*_error_ = 223590.39, *p* = 0.70, *N.S*]; Perspective Taking was associated with accuracy [*F*_(1, 8)_ = 6.56, *MS*_error_ = 0.034, *p* = 0.034], but not with *RT* [*F*_(1, 8)_ = 0.067, *MS*_error_ = 223590.39, *p* = 0.80, *N.S*]; Empathic Concern was inversely associated with accuracy [*F*_(1, 8)_ = 6.69, *MS*_error_ = 0.032, *p* = 0.032], but not with *RT* [*F*_(1, 8)_ = 0.94, *MS*_error_ = 223590.39, *p* = 0.36, *N.S*]; and Personal Distress was not associated with either accuracy [*F*_(1, 8)_ = 0.76, *MS*_error_ = 0.034, *p* = 0.41] or *RT* [*F*_(1, 8)_ = 0.085, *MS*_error_ = 223590.39, *p* = 0.78, *N.S*].

These results suggest that while the accuracy of giving food or water to a hungry or thirsty child was not dependent on the child's feedback, it was dependent on three out of four dimensions of dispositional empathy. The cognitive dimensions (Perspective Taking and Fantasy) were associated with increased accuracy and one affective dimension (Empathic Concern) was associated with decreased accuracy. Age also played a role in accuracy and thus was included as a covariate in the neuroimaging analyses below.

### PDMT fMRI results

#### Empathy-related neuroimaging results

**Figure 2:**
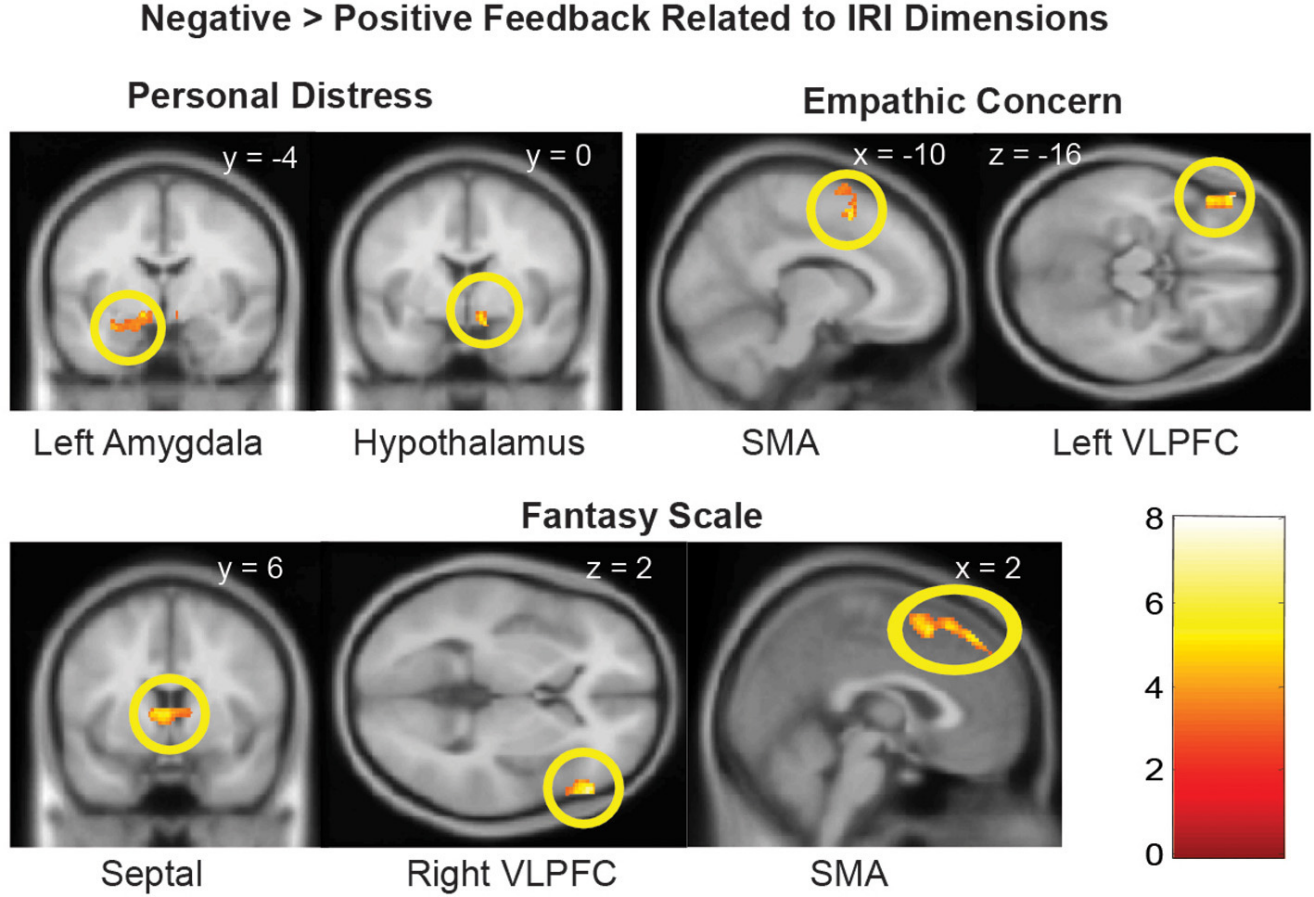
**Brain regions with Negative > Positive Feedback differential response that were associated distinct dimensions of dispositional empathy measured with IRI**. Referring to Table 3 for the coordinates, number of voxels, Z-score, and *p*-values. The clusters in the statistical brain map were presented at *p* = 0.005, uncorrected, with the color map in T-scores.

We next examined whether the empathy-dependent ROIs were sensitive to children's distress vs. non-distress as a function of each distinct dimension of dispositional empathy. To do so, we conducted a Negative vs. Positive Feedback (all children combined) general linear model with one dimension of dispositional empathy at a time as a regressor, and age as a covariate. The results are summarized in Table 3 and illustrated in Figure 2.

**Table 3:** **Empathy-related Neural Responses in Negative vs. Positive Feedback**.

**Brain region**	**Side**	**MNI coordinates**			**No. of voxels**	***Z* score**
	***X***	***Y***	***Z***		
**PERSONAL DISTRESS, POSITIVE ASSOCIATION**						
Amygdala^a^	L	−30	−2	−18	48	3.36
Hypothalamus^a^	R	8	0	−12	16	3.60
**PERSONAL DISTRESS, NEGATIVE ASSOCIATION**						
None						
**EMPATHIC CONCERN, POSITIVE ASSOCIATION**						
SMA^a^	L	−10	10	54	159	4.06
VLPFC^a^	L	−50	40	−14	202	4.20
**EMPATHIC CONCERN, NEGATIVE ASSOCIATION**						
None						
**FANTASY SCALE, POSITIVE ASSOCIATION**						
Septal area^a^	L/R	−4	4	6	38	3.77
SMA^a^	R	2	14	15	144	3.91
VLPFC^a^	R	56	32	2	319	4.21
**FANTASY SCALE, NEGATIVE ASSOCIATION**						
None						
**PERSPECTIVE TAKING, POSITIVE OR NEGATIVE ASSOCIATION**						
None						

a *Family-wise error corrected in the ROI at p < 0.05*.

#### Cortisol-related neuroimaging results

**Figure 3:**
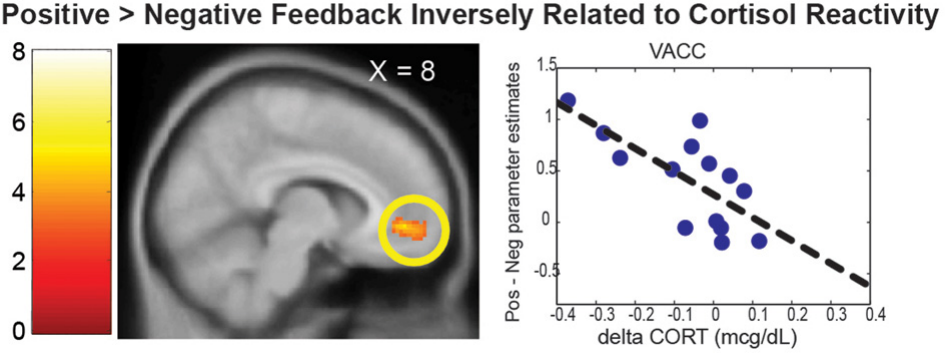
**The VACC was differentially activated by the Positive > Negative Feedback**. The scatter plot depicted the mean parameter estimate of the VACC cluster on the Y-axis and the cortisol reactivity (dCORT) on the X-axis. The cluster in the statistical brain map was presented at *p* = 0.005, uncorrected, with the color map in T-scores.

In this section, neural correlates of cortisol reactivity (dCORT) during the PDMT were identified based on the two-step approach described in the methods. First, consistent with the literature, the VACC that mediates face-based values (Ho et al., 2012) was differentially activated by Positive vs. Negative Feedback during the task [*k* = 452 voxels, peak at (8, 46, −4), *Z* = 3.63, *p* = 0.035, s.v.c.]. This was equivalent to being differentially de-activated by the Negative vs. Positive Feedback. In addition, the Positive vs. Negative Feedback differential response in the VACC was inversely correlated with cortisol reactivity, dCORT, (*r* = −0.65, *p* = 0.022, *df* = 10), controlling for age and time of scan (Figure 3). These results suggested that the more discrimination between positive and negative signals in social reward as mediated by the VACC, the less cortisol reactivity was observed.

**Figure 4:**
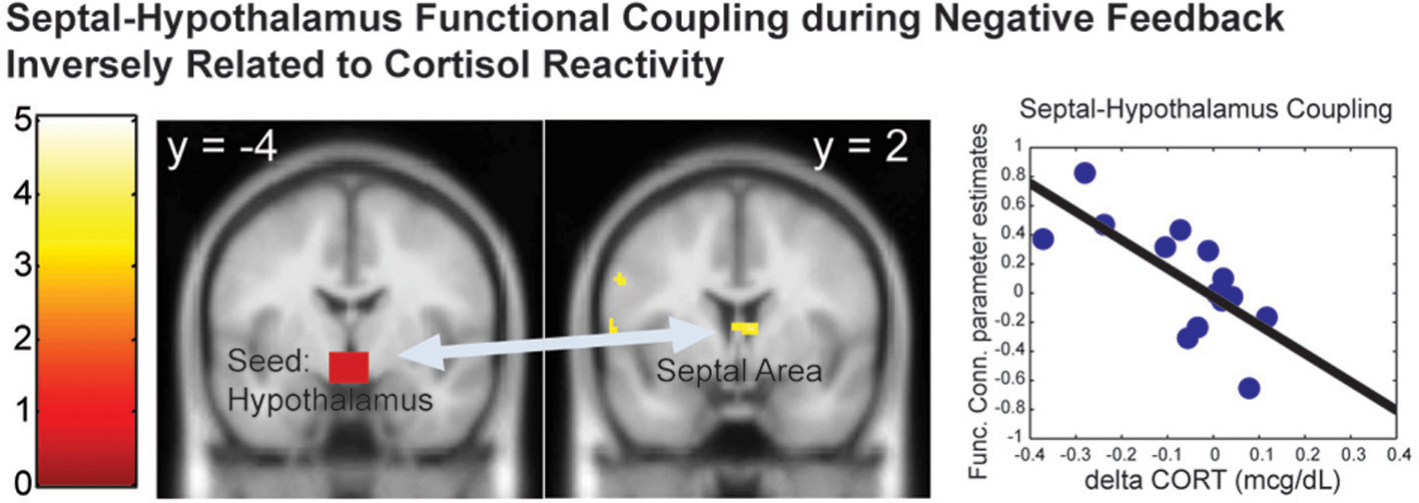
**The functional connectivity between the hypothalamus-septal area during the Negative Feedback was inversely correlated with the cortisol reactivity (dCORT)**. The cluster in the statistical brain map was presented at *p* = 0.005, uncorrected, with the color map in T-scores.

Since the hypothalamus is the final central mechanism in the brain that mediates peripheral cortisol responses, we examined the functional connectivity with the hypothalamus as a function of the cortisol reactivity during the distressed condition (the negative feedback across all children). We found that the functional coupling between the hypothalamus and the septal area [*k* = 37 voxels, peak at (6, 2, 8), *Z* = 3.24, *p* = 0.019, s.v.c.] during the Negative Feedback across all children was inversely correlated with dCORT (*r* = −0.60, *p* = 0.038, *df* = 10), controlling for age and time of scan (Figure 4). These results suggested that positive coupling between the septal area and hypothalamus was related to cortisol reduction, consistent with the septal area's role in stress-regulation (Singewald et al., 2011) and human empathy (Morelli et al., 2014) in the literature.

## Discussion

In the current study, we examined the roles of four dimensions of dispositional empathy in stress reactivity during a social evaluation task in healthy women (Part 1). We also examined the interplay between brain function, dispositional empathy, and cortisol reactivity to negative child feedback among mothers participating in a parental decision making task (Part 2).

In Part 1, we found that the Personal Distress dimension of dispositional empathy was associated with increased cortisol reactivity while participants were preparing a speech in the Social Evaluation Test (Wager et al., 2009), and Perspective Taking was associated with decreased cortisol reactivity. These results suggest that dispositional empathy may play a generalized role in people's stress response in a social context, even when the context was not necessarily empathy-related. Thus, trait Personal Distress may be related to chronic hyper-reactivity in the limbic-hypothalamus-pituitary-adrenal (LHPA) axis, similar to other self-focused traits (e.g., Edelstein et al., 2010; Reinhard et al., 2012).

In Part 2, the behavioral results of the Parental Decision Making Task suggested that the Perspective Taking and Fantasy Scale dimensions were associated with greater accuracy, while Empathic Concern was associated with less accuracy. Personal Distress was the only dimension that was not associated with the accuracy of the choice responses. However, none of the four dimensions of dispositional empathy were associated with response times in this task. Since Empathic Concern was positively correlated with both Perspective Taking and Fantasy (see Table 2), the meaning of these dimensions' associations with accuracy is unclear, and will require further examination in the future.

In the neuroimaging results of Part 2, we examined whether the four dimensions of dispositional empathy were related to neural activation in the amygdala and hypothalamus as part of the LHPA-axis (Feldman et al., 1995; Dayas et al., 1999; Wilkinson and Goodyer, 2011). We found that the Personal Distress was the only empathy subscale that was associated with greater hypothalamus and left amygdala responses to negative (vs. positive) feedback from the children. These results suggest that mothers with greater tendencies to experience vicarious distress may have increased reactivity in the limbic-hypothalamus end of the LHPA-axis during parental care tasks. If so, this is consistent with the cortisol reactivity results as reported in Part 1, which indicates a consistent relationship between Personal Distress and the LHPA-axis reactivity across two different contexts.

Both Empathic Concern and Fantasy were associated with more Negative vs. Positive Feedback activation in the SMA and VLPFC, but the clusters were lateralized differently on the left hemisphere, for Empathic Concern, and the right hemisphere, for Fantasy. The distinct lateralization related to the two dimensions of empathy implicated that the interplay between the relatively more verbal left hemisphere and more non-verbal right hemisphere may contribute to the distinct dimensions of dispositional empathy.

In addition, the septal area was differentially activated by Negative vs. Positive Feedback as a function of the Fantasy subscale only. The engagement of the septal area may help mothers buffer stress by regulating the hypothalamus in the LHPA-axis, since the functional coupling between the septal area and hypothalamus was found to be inversely associated with cortisol reactivity during negative feedback. In addition, since the septal area has been implicated to play a role in empathy as part of a prosocial motivation system (Morelli et al., 2014), these results suggest that the propensity to identify with other persons, as indexed by the Fantasy subscale, may facilitate the engagement of prosocial motivation in response to others' distress by engaging the septal area. In turn, this increased septal area signaling to the hypothalamus may down-regulate stress-related cortisol reactivity.

In addition to the prosocial motivation, the hypothalamus-dependent cortisol response may also be buffered by social reward processes. We found that the VACC mediated the valuation of the face-based reward, as it was differentially activated by the positive feedback as compared to the negative feedback. This is consistent with the role of VACC in different aspects of social reward (Bolling et al., 2011; Ho et al., 2012) and self-referential processing of emotional stimuli (Yoshimura et al., 2014). It is also consistent with VACC response to positive vs. negative feedback from peers in an evaluative social feedback experiment (Somerville et al., 2010). Moreover, the degree of such activation was related to decreased cortical reactivity. These results suggest that reduced cortisol reactivity may result from better attunement between mothers' social reward valuations, mediated by the VACC, and the emotional signals in children's feedback.

### Strengths, limitations, and future directions

This study examines the relationship between dispositional empathy and stress regulation, in different contexts—both in a general socially evaluative context, but also in a parenting context. While it has been reported that, using similar methodologies in a pre- and post-fMRI task design, the salivary cortisol reactivity to a non-stress-inducing fMRI task can be associated with trait anxiety independent of the task (Tessner et al., 2006), to our knowledge the current study is the first to examine the relationship between the salivary cortisol and brain responses during a personally significant but not stress-inducing task as a function of empathy dimensions. Although it is limited by both its sole consideration of women only and its small sample size, it can pave the way to additional future research on more general and larger samples. For example, it would be interesting to see if our effects are replicated among males, and particularly among fathers. If so, this would point to a generalized caregiving system that has evolved beyond maternal care to help regulate stress responses of any type of giver. Future research should also examine whether the four dimensions of empathy are associated with non-social stress regulation (e.g., doing math problems) rather than just social stressors as examined in the current study.

## Conclusion

Consistent with the Caregiving Model of Stress Regulation (Swain et al., 2012, 2013), this study provides some preliminary evidence that the dispositional empathy may be associated with stress regulation. More empathic and attuned (i.e., other-oriented) parents have been shown to positively influence their child's developmental trajectories (Landry et al., 2006). Considering this, interventions designed to increase parental empathy, e.g., (Konrath et al., 2014), may be beneficial to both the children and the parents themselves.

### Conflict of interest statement

The authors declare that the research was conducted in the absence of any commercial or financial relationships that could be construed as a potential conflict of interest.
